# A clinical study on relationship between visualization of cardiac fibroblast activation protein activity by Al^18^F-NOTA-FAPI-04 positron emission tomography and cardiovascular disease

**DOI:** 10.3389/fcvm.2022.921724

**Published:** 2022-08-22

**Authors:** Zhehao Lyu, Wei Han, Hongyue Zhao, Yuying Jiao, Peng Xu, Yangyang Wang, Qiuyi Shen, Shuai Yang, Changjiu Zhao, Lin Tian, Peng Fu

**Affiliations:** ^1^Department of Nuclear Medicine, The First Affiliated Hospital of Harbin Medical University, Harbin, China; ^2^Department of Pathology, The First Affiliated Hospital of Harbin Medical University, Harbin, China

**Keywords:** diabetes mellitus, fibroblast, myocardial infarction, risk factors, tomography

## Abstract

**Objective:**

FAP plays a vital role in myocardial injury and fibrosis. Although initially used to study imaging of primary and metastatic tumors, the use of FAPI tracers has recently been studied in cardiac remodeling after myocardial infarction. The study aimed to investigate the application of FAPI PET/CT imaging in human myocardial fibrosis and its relationship with clinical factors.

**Materials and methods:**

Retrospective analysis of FAPI PET/CT scans of twenty-one oncological patients from 05/2021 to 03/2022 with visual uptake of FAPI in the myocardium were applying the American Heart Association 17-segment model of the left ventricle. The patients’ general data, echocardiography, and laboratory examination results were collected, and the correlation between PET imaging data and the above data was analyzed. Linear regression models, Kendall’s TaU-B test, the Spearman test, and the Mann–Whitney *U* test were used for the statistical analysis.

**Results:**

21 patients (60.1 ± 9.4 years; 17 men) were evaluated with an overall mean LVEF of 59.3 ± 5.4%. The calcific plaque burden of LAD, LCX, and RCA are 14 (66.7%), 12 (57.1%), and 9 (42.9%). High left ventricular SUVmax correlated with BMI (*P* < 0.05) and blood glucose level (*P* < 0.05), and TBR correlated with age (*P* < 0.05). A strong correlation was demonstrated between SUVmean and CTnImax (*r* = 0.711, *P* < 0.01). Negative correlation of SUVmean and LVEF (*r* = −0.61, *P* < 0.01), SUVmax and LVEF (*r* = −0.65, *P* < 0.01) were found. ROC curve for predicting calcified plaques by myocardial FAPI uptake (SUVmean) in LAD, LCX, and RCA territory showed AUCs were 0.786, 0.759, and 0.769.

**Conclusion:**

FAPI PET/CT scans might be used as a new potential method to evaluate cardiac fibrosis to help patients’ management further. FAPI PET imaging can reflect the process of myocardial fibrosis. High FAPI uptakes correlate with cardiovascular risk factors and the distribution of coronary plaques.

## Introduction

Cardiovascular disease (CVD) is rapidly becoming a global health problem, with an increasing incidence in low-income countries (1). The ability to identify CVD-prone individuals depends on understanding and detecting risk factors (1). In the INTERHEART Study, a case-control study of acute myocardial infarction, participants were recruited from 52 low-, middle-, and high-income countries in South and Southeast Asia, Africa, China, Japan, Europe, and the Middle East, Australia/New Zealand, North and South America (2) demonstrated nine easily assessed and common traditional risk factors which were statistically associated with an increased risk of myocardial infarction, including tobacco smoking, dyslipidemia, hypertension, diabetes, abdominal obesity, and psychosocial factors (2). It is urgent that a reliable imaging technique is developed which can quantify the impact of these common risk factors on cardiac remodeling. Fibroblasts play a crucial role in cardiac tissue remodeling and wound healing (3). One significant characteristic of activated cardiac fibroblasts is the expression of fibroblast activation protein (FAP) (4). After myocardial infarction, FAP is strongly expressed compared with resting fibroblasts (5). Lindner et al. developed a tracer for PET scans that targets FAP in 2018. The tracer consists of a quinolone-based FAP inhibitor (FAPI) labeled with a radio nucleoid, which reliably binds and stains FAP (6). It has been proven to be useful in a variety of tumor imaging applications (7). In previous studies, FAPI-04 has been shown to be a promising radiotracer of post-MI fibroblast activation (5, 8–11), or may provide a novel biomarker of left ventricular remodeling that is complementary to existing techniques such as MRI (12, 13). In this study, we aimed to compare FAPI tracer accumulation and the benefit of FAPI PET/CT scans in patients with various CVD risk factors and whether can assist in evaluating the degree of fibrosis of coronary artery plaques.

## Materials and methods

### Patients

A total of 21 of 159 oncological patients with increased myocardial FAPI uptake who had undergone Al^18^F NOTA FAPI-04 PET were retrospectively analyzed. All patients gave written informed consent to undergo FAPI PET/CT following the German Pharmaceuticals Act § 13(2b) regulations. The clinical translational study of Al^18^F-NOTA-FAPI-04 was approved by the Ethics Committee (approval no. 2021XJSS01) and registered in the Chinese Clinical Trial Registry (ChiCTR2100051406). All patients enrolled in this study signed written informed consent forms. We assessed clinical features, including cardiovascular risk factors, imaging parameters from echocardiography, and a broad laboratory panel. Only patients who had a contraindication to undergo PET/CT (e.g., pregnant women and women of childbearing age) or patients who refused the procedure did not undergo imaging. All reported investigations were conducted by the Declaration of Helsinki and with the national regulations.

### Radiotracer synthesis

The synthesis and labeling of Al^18^F-NOTA-FAPI-04 have already been described previously (14). Following the Drug Administration Law of the People’s Republic of China, indication and labeling of the FAPI-tracers were conducted under the physician’s direct responsibility. Injected activities were dependent on labeling yields. Synthesis and labeling of FAPI-04 have already been described previously (6) –an effective dose of 1.6 mSv/100 MBq—an upper limit of 370 MBq regarding radiation exposure and a lower limit of 100 MBq per exam to achieve a sufficient count rate have been considered (15).

### PET/CT image acquisition

No patients were required to fast, and venous blood glucose levels were not controlled. Radioactivity ranging from 129.5 to 148 MBq of Al^18^F-NOTA-FAPI-04 isotope (Jiangyuan Industrial technology trade Co., Ltd., Jiangsu, China, radiochemical purity > 95%) was intravenously injected. After urinating in quiet, light-avoidance conditions (15 min), the PET/CT images were acquired using a 16-slice Gemini GXL PET/CT scanner (Philips Medical System). A low-dose CT scan (tube voltage: 120 kV, tube current: 50 mAs, slice thickness: 5.0 mm, pitch: 1.0) was acquired for attenuation correction, and then the PET images were acquired (1.5 min per bed position, 6–7 PET bed positions). According to the agency’s standard clinical protocols, the scan range was from the head to the mid-thigh. The line of response reconstruction algorithm was used to reconstruct the image without post-reconstruction filtering after automatic random and scattering correction.

### Image evaluation

PET data were analyzed by two nuclear medicine specialists (Zhao CJ, Fu P) on a consensus decision who were board-certified. Myocardial tracer uptake was quantified as SUV_max_, SUV_mean_, and target to background ratio (TBR) from static images 15 min after tracer injection. The background (blood pool, right atrium) was quantified using a circular 1-cm-diameter sphere. Tracer-uptake patterns in axial images were assessed according to the 17-segment model of the American Heart Association blinded for the affected coronary vessels (culprit lesions). The left ventricle is divided into three areas: a. LAD area including Seg. 1, 2, 7, 8, 13, 14, and 17; b. LCX area including Seg. 5, 6, 11, 12, and 16; c. RCA area including Seg. 3, 4, 9, 10, and 15.

### Statistical analysis

Statistical analyses were performed using SPSS software version 25.0 (SPSS, Chicago, IL, United States), GraphPad Prism (version 8.4.2; GraphPad Software, San Diego, CA, United States), and the *R* language (version 3.6.3^1^). Quantitative values were expressed as mean ± SD or median and appropriate range, and categorical variables were presented as a rate or percentage. Shapiro–Wilk test for continuous variables shows that all continuous variable data do not meet normal distribution. A comparison of non-parametric data was performed using a Wilcoxon test. For correlation analyses, Kendall’s TaU-B test was used to test the correlation between categorical and continuous variables. The Spearman test was used to test the correlation between continuous and continuous variables. THE Mann–Whitney *U* test was used for univariate analysis of continuous and categorical variables. All statistical tests were performed 2-sided, and *P* < 0.05 indicated statistical significance.

## Results

### Patients’ characteristics

**FIGURE 1:**
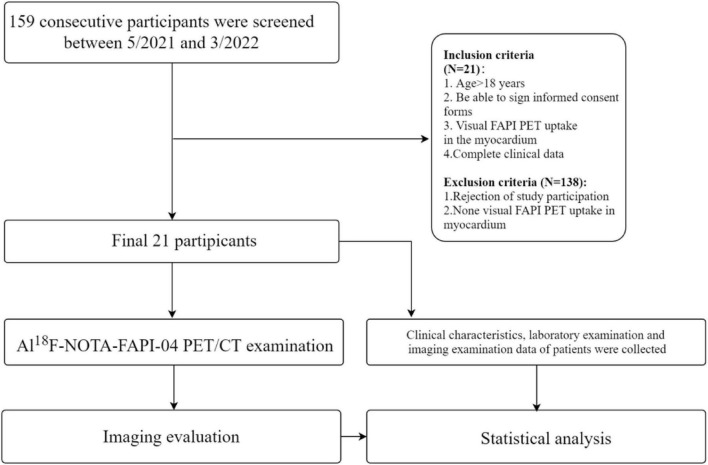
Study flow chart. Patients were consecutively enrolled.

Detailed characteristics are presented in Table 1. From 05/2021 to 03/2022, *N* = 21 of 159 patients underwent PET imaging for staging different kinds of cancers or for definitive diagnosis with visual uptake of FAPI in the myocardium (Figure 1). The majority of patients were male (17/21, 81.0%), with an overall mean age of 60.1 ± 9.4 years at the time of the PET scan. The overall mean left ventricular ejection fraction (LVEF) is 59.3 ± 5.4%. Median levels of cardiac troponin I (CTnI) at admission and peak creatine kinase isoenzyme (CKMB) were 0.03 ng/ml (75th percentile > 0.5 ng/ml) and 1.1 ng/ml (75th percentile > 9.5 ng/ml), respectively. The calcific plaque burden of LAD, LCX and RCA are 14 (66.7%), 12 (57.1%), and 9 (42.9%), respectively.

**TABLE 1:** Patient characteristics (*n* = 21 patients).

Characteristics	Values
Male, n (%)	17 (81.0)
Age at FAPI scan, mean ± SD, years	60.1 ± 9.4
LVEF, mean ± SD, (%)	59.3 ± 5.4
**Cardiovascular risk factors**	
Diabetes, n (%)	12 (57.1)
HTN, n (%)	11 (52.4)
Current smoker, n (%)	11 (52.4)
Current drinker, n (%)	10 (47.6)
History of CAD, n (%)	11 (52.4)
BMI, mean ± SD, kg/m^2^	22.2 ± 3.5
**Blood Results**	
Median (interquartile range) peak cTnI, ng/ml	0.03 (0.5)
Median (interquartile range) peak CK, U/l	97.7 (309.3)
Median (interquartile range) peak CKMB, ng/ml	1.1 (9.5)
Median (interquartile range) peak Hs-CRP, mg/L	11.8 (79.1)
Median (interquartile range) peak TG, mmol/L	1.37 (2.42)
**Vessels of calcific plaques, n (%)**	
LAD	14 (66.7)
LCX	12 (57.1)
RCA	9 (42.9)

### Visual and quantitative assessment of myocardial FAPI uptake in the overall cohort

**FIGURE 2:**
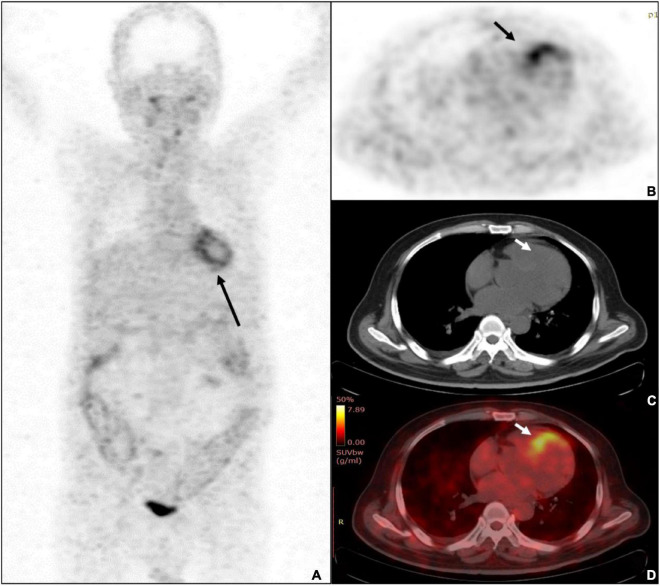
A 68-years-old male patient was diagnosed with ulcerative colitis and diabetes mellitus. **(A)** The whole-body MIP of Al^18^F-NOTA-FAPI-04 PET imaging demonstrated diffused uptake pattern in LV (black arrow) and diffused uptake with colons. **(B)** The PET imaging showed the highest uptake of SUVmax was 7.0 (black arrow). **(C)** Unfused CT image of the heart. **(D)** The fusion imaging showed the septal wall of prominent uptake of FAPI-04.

FAPI imaging demonstrated moderate to intense myocardial uptake on visual interpretation in all twenty-one patients. All patients had diffused or focal uptake in the left ventricular (LV) myocardium [21/21 (100%)], and five patients also demonstrated tracer uptake in the right ventricular (RV) wall. There were three distinct patterns: diffuse, focal on diffuse, and focal. 6 out of twenty-one patients (28.6%) demonstrated focal myocardial FAPI uptake above background, 4 patients (19.0%) demonstrated diffused FAPI uptake, and the other 11 patients (52.4%) demonstrated focal on diffused FAPI uptake. The highest SUV_max_, SUV_mean,_ and TBR were found at 15 min with 7.0, 3.1, and 7.2, respectively (Figure 2). Average uptake showed an SUV_max_ of 4.4 ± 1.2 (range, 2.6–7.0), SUV_mean_ of 2.0 ± 0.5 (range, 1.2–3.1), TBR of 3.5 ± 1.3 (range, 1.8–7.2).

### Signal intensity correlates with patient characteristics and cardiovascular risk factors

**FIGURE 3:**
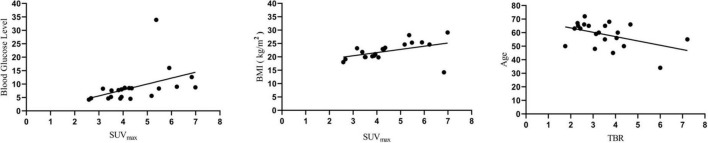
Linear regression analysis showed that blood glucose level and BMI were positively correlated with SUV_max_, while age was negatively correlated with TBR.

**FIGURE 4:**
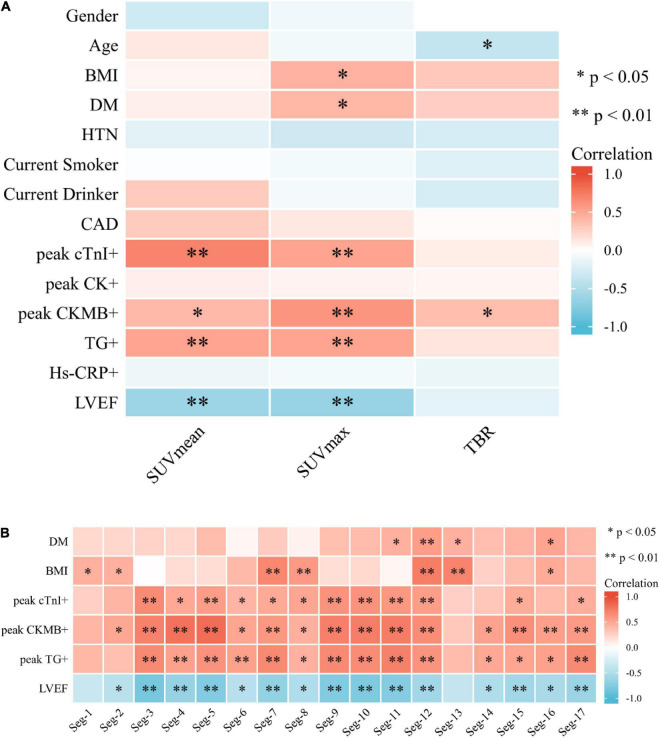
**(A)** Heat map of cardiovascular risk factors, blood tests, and left ventricular ejection fraction associated with SUV uptake. BMI and DM demonstrated a correlation with SUVmax (*P* < 0.05). Age showed a negative correlation with TBR (*P* < 0.05). **(B)** Heat map of correlation analysis among SUVmax value of 17-segment of the left ventricle with DM, BMI, blood test, and LVEF.

In the linear regression analysis, high left ventricular SUV_max_ correlated with BMI (*P* < 0.05) and blood glucose level (*P* < 0.05), and TBR correlated with age (*P* < 0.05) (Figure 3). Correlation analysis showed a significant correlation between BMI and FAPI uptake (SUVmax) (*r* = 0.44, *P* < 0.05), diabetes mellitus and FAPI uptake (SUVmax) (*r* = 0.44, *P* < 0.05), age and FAPI uptake (TBR) (*r* = −0.38, *P* < 0.05) (Figure 4A). However, there was no significant difference in SUV_max_ between BMI normal group and BMI overweight group (3.7 vs. 5.5, *P* = 0.5); diabetes mellitus group, and non-diabetes mellitus group (4.9 vs. 3.6, *P* = 0.26), TBR between age > 60 group and age < 60 group (2.9 vs. 4.0, *P* = 0.3). There was also no significant difference in FAPI uptake (SUVmean) in any cardiovascular risk factor groups.

### Association of myocardial FAPI uptake with a blood test and left ventricular ejection fraction

Strong correlation was demonstrated between SUV_mean_ and CTnI_max_ (*r* = 0.711, *P* < 0.01), followed by a moderate correlation between SUV_mean_ and CKMB_max_, TG_max_, respectively (*r* = 0.41, *P* < 0.05; 0.53, *P* < 0.01) (Figure 4A). There was also a moderate correlation between SUV_max_ and CTnI_max,_ CKMB_max_, and TG_max_ (*r* = 0.53, 0.61, and 0.53, all *P* < 0.01) (Figure 4A). TBR was only weakly related to CKMB_max_ (*r* = 0.38, *P* < 0.05) (Figure 4A). There was no significant correlation between other PET parameters and myocardial injury markers. Negative correlation of SUV_mean_ and LVEF (*r* = −0.61, *P* < 0.01), SUV_max_ and LVEF (*r* = −0.65, *P* < 0.01) were found, indicating a moderate inverse relation between those 3 measurements. The multiple linear regression showed CKMB and CTnI were independent risk factors for increased SUV_max_ (R^2^ = 0.676, *P* < 0.01) and SUV_mean_ (R^2^ = 0.690, *P* < 0.01), respectively.

### Association of myocardial FAPI uptake in 17-segment model with DM, BMI, blood test and left ventricular ejection fraction

We noticed a significantly higher FAPI uptake in the septal than the lateral segments (3.05 vs. 2.58, *P* = 0.005) when analyzing FAPI SUV_max_ uptake in the 17-segment model of the LV. In the correlation analysis, SUV_max_ of the Seg. 11, 12, 13, and 16 correlated with the DM (*r* = 0.46, 0.56, 0.46 and 0.53, all *P* < 0.05); SUV_max_ of the Seg. 1, 2, 7, 8, 12, 13, and 16 correlated with the BMI (*r* = 0.46, 0.44, 0.68, 0.58, 0.74, 0.71 and 0.49, all *P* < 0.05). A strong correlation was shown among SUV_max_ of most segments of LV, CTnI_max,_ CKMB_max_, TG_max_, and LVEF (Figure 4B).

### The relationship between the myocardial FAPI uptake and calcific plaques of culprit vessel territory

**FIGURE 5:**
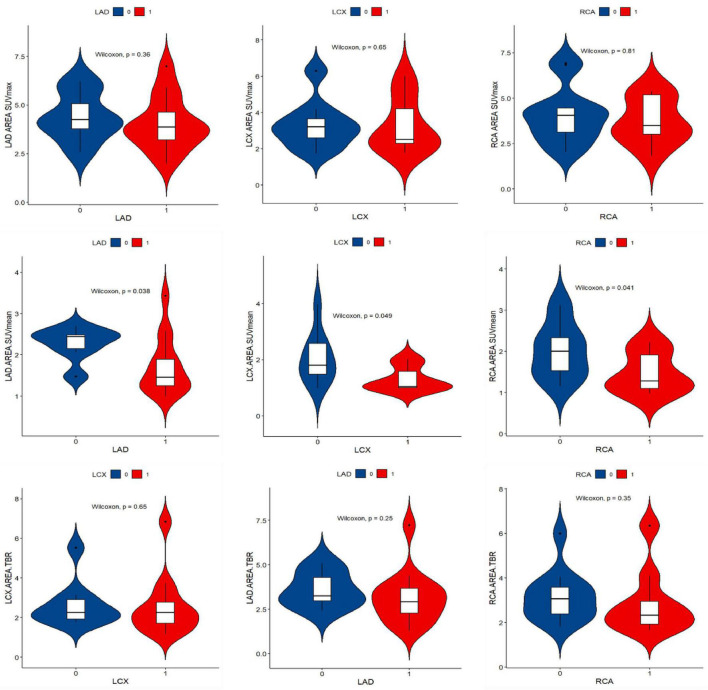
Violin plot of univariate analysis of FAPI uptake difference in left ventricular calcification and non-calcification region. “1” stands for the calcific area, “0” stands for the non-calcific area. SUV_mean_ demonstrated higher FAPI uptake in non-calcific areas than calcific areas.

**FIGURE 6:**
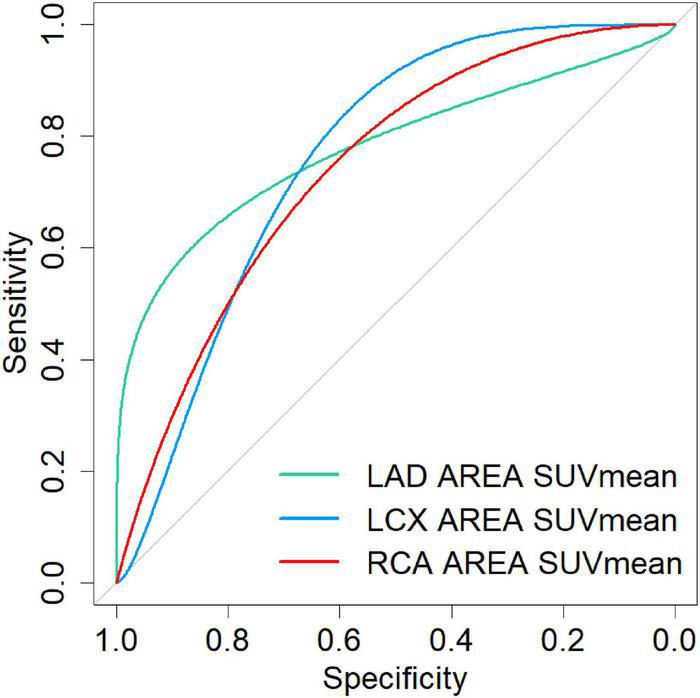
The ROC curve for predicting calcified plaques by myocardial FAPI uptake (SUV_mean_) in LAD, LCX, and RCA territory showed areas under the curve (AUCs) were 0.786 (95%CI: 0.581–0.99), 0.759 (95%CI: 0.521–0.998), and 0.769 (95%CI: 0.559–0.978), respectively.

The numbers of LAD, LCX, and RCA affected by calcified plaques were 14, 12, and 9. In the univariate analysis, the SUV_mean_ of LAD, LCX, and RCA non-calcific areas showed significantly higher than those of calcific areas (2.27 vs. 1.72, 2.06 vs. 1.31, 2.02 vs. 1.47, all *P* < 0.05) (Figure 5). ROC curve for predicting calcified plaques by myocardial FAPI uptake (SUV_mean_) in LAD, LCX, and RCA territory showed areas under the curve (AUCs) were 0.786 (95%CI: 0.581–0.99), 0.759 (95%CI: 0.521–0.998), and 0.769 (95%CI: 0.559–0.978), respectively (Figure 6). The SUV_mean_ cutoff values of LAD, LCX, and RCA areas were 1.988, 1.257, and 1.438, respectively. The accuracy, sensitivity, and specificity of LAD, LCX, and RCA areas were showed in Table 2. The ROC curve for predicting calcified plaques by myocardial FAPI uptake (SUV_max_ and SUV_TBR_) in LAD, LCX, and RCA territory were showed in Supplementary Figures A, B.

**TABLE 2:** Comparison of the difference in predictive ability between the calcification and the SUVmean FAPI uptake.

	ACC	SEN	SPE	Cutoff	AUC	AUC.SE	*P-value*
LAD	0.81	0.857	0.786	1.988	0.786	0.104	0.019
LCX	0.762	0.889	0.667	1.257	0.759	0.122	0.025
RCA	0.762	0.833	0.667	1.438	0.769	0.107	0.02

## Discussion

Due to the lack of functional imaging techniques, early detection of CVD has been unsatisfactory. A cardiac biopsy is considered as the gold standard, but a diagnosis is not immediately available. A biopsy can be falsely negative if there is the patchy distribution of the pathology (16). Thus, myocardial fibrosis needs to be evaluated non-invasively to monitor the process of myocardial fibrosis. A fibroblast activation protein (FAP) is an atypical type II transmembrane serine protease with both endopeptidase and post-proline dipeptidyl peptidase activity (17). Activated FAP is almost exclusively found in wound healing and pathological conditions such as scar formations (18), liver cirrhosis (19), inflammation (20) and cancer (21).

Researchers conducted preclinical studies showing that anti-fibrosis therapy inhibits static fibroblast activation and facilitates fibroblast interconversion, resulting in improved left ventricular function (22–26). A promising field of active research targets the immune system to benefit injured hearts (24). Transferring T cells expressing a chimeric antigen receptor against fibroblast activation protein results in a significant reduction of cardiac fibrosis and restoration of cardiac function after injury in mice (23). After the coronary injury, Varasteh et al. showed that ^68^Ga-FAPI uptake peaked at day 6 post-MI, most of which occurred in the border-ischemic area (5). In contrast, some results suggest that FAP is not crucial for cell proliferation, adherence and migration within the myocardium after MI (8). The lack of non-invasive tools that can monitor fibroblast activation in humans, however, has limited the success of translating these results to human patients. This is due to the lack of non-invasive tools that can monitor fibroblast activation in patients (5). Specifically, myofibroblasts play a beneficial role in the adverse effects of the long-term increase in reactive fibrosis, and the timing of safe and effective anti-fibrotic therapy needs to be carefully selected. Therefore, diagnostic strategies aimed at detecting active myofibroblasts could better understand their presence in damaged myocardium and evaluate the efficacy of anti-fibrosis therapies (9, 10).

For the first time, this study describes cardiac FAPI uptake patterns in patients undergoing PET imaging with Al^18^F-NOTA-FAPI-04. As the most studied and reported FAPI tracer, ^68^Ga-FAPI-04 has the highest level of PET molecular imaging research, but it is limited in its batch activity (21, 27). Al^18^F-NOTA-FAPI-04 is a promising alternative that combines the advantages of a chelator-based radiolabeling method with the unique properties of fluorine-18 (27). Under convenient manual operation, Al^18^F-NOTA-FAPI can be achieved with high radiolabeling yields and specific activities (14). This study reports an association between FAPI uptake and risk factors for cardiovascular diseases. Diabetes mellitus, overweight, and aging were associated with increased FAPI uptake, suggesting metabolic changes and cardiac FAP activation might be related.

A major phenotypic characteristic of cardiac fibroblasts that respond to stimulation is increased proliferation in the myocardium. Cardiac FAP activation has been reported in a variety of pathological conditions (3, 28–30). Myocardial fibrosis after myocardial infarction has been reported in several studies (5, 9, 11, 31). Radiotracer delivery may be inadequate in infarcted areas, explaining this pattern of tracer uptake. There is a need for these studies to assess whether FAPI can image the reaction of fibroblasts during coronary intervention. As reported by Nagaraju et al. in an MI pig model, upregulated expression of TGF-β1, a strong stimulator for fibroblast differentiation and positive FAP-α staining, was seen in the border and remote myocardium in addition to the scar region, suggesting that the activation of fibroblasts can occur in the non-infarcted area (28).

According to our study, myocardial fibrosis can still be detected by FAPI imaging under cardiovascular risk factors even in the absence of coronary artery occlusion, providing more evidence for clinical decision-making. As part of our current research on cardiac tracer accumulation in humans, we found that approximately 13.2% (21/159) of individuals in this cohort showed cardiac tracer accumulation in an unselected population. These results suggest that FAPI PET/CT imaging may assist in the visualization of myocardial injury. Accumulation of FAPI is likely to be caused by prior myocardial injury associated with CVD. FAPI accumulation revealed that patients with FAPI accumulation were older and more likely to have diabetes mellitus, obesity and lower LVEF. This is consistent with other studies’ data suggesting that LV function (32, 33), age (34), and especially DM (35) are associated with myocardial fibrosis.

Our study showed that SUV_max_ values were positively correlated with blood glucose and BMI, but not SUV_mean_ or TBR values. This might be because SUV_max_ measured the maximum value of the region of interest, which was higher than the average value, and achieved an excellent observation. TBR values were negatively correlated with age, which may indicate that the progression of myocardial fibrosis is gradually declining with the prolongation of the disease, thus affecting the uptake of FAPI. Interestingly, however, SUV_max_ and SUV_mean_ did not show a meaningful correlation, which reflects the importance of FAPI-PET multi-parameter analysis, and further observation is still needed in follow-up large-sample studies. Patients with multiple risk factors showed a greater increase than those with a single risk factor. FAPI enrichment is a hallmark of metabolic diseases such as diabetes and obesity (36). Both diabetes mellitus and transaortic constriction promote cardiomyocyte hypertrophy and excessive cardiac fibrosis based on animal studies (37, 38). According to these models, cardiac fibrosis is caused by activated inflammatory pathways and TGFβ/Mothers Against Decapentaplegic Homolog Protein signaling (8, 35). There may be a simultaneous dysregulation of metabolic and hypertrophic pathways in the human heart responsible for the increase in FAPI uptake. Previous studies demonstrated circulating FAP and TGF-β1 levels significantly declined (31). The inconsistency between tissue-level and circulating-level biomarkers raises the possibility that peripheral biomarkers may not be reliable indicators of tissue status (39).

Additionally, we demonstrated that FAPI uptake exhibits an inverse relationship with left ventricular systolic function, as well as a positive relationship with maximum CTnI_max_, CKMB_max_, and TG_max_, reflecting the extent of myocardial damage. Kessler L et al. also demonstrate a strong inverse correlation between the amount of activated fibroblasts and left ventricular systolic function as well as a very strong positive correlation between the amount of activated fibroblasts and maximum CK reflecting the extent of myocardial damage (11). A minimal change in LVEF may accompany progressive fibrosis. It can help with identifying a high-risk cohort and individuals with DCM (40). This suggests that visualization of FAP expression by FAPI PET/CT imaging could help determine the degree of myocardial damage following DM in progressive CAD and other chronic myocardial diseases. A strong correlation between the FAPI uptake, the peak cTnI/CKMB/TG serum levels, and the LVEF in our study demonstrates that FAPI PET/CT might be an accurate assessment of myocardial damage. The serum levels of cardiomyocyte injury markers (LDH, CK-MB, and CTnI), TG, and TC were also increased in CHD rats (41). This finding may lead to the potential of the FAPI PET imaging method to assist in hazard risk stratification in CVD patients (11).

Different extracellular matrix components have been evaluated for molecular imaging of cardiac fibrosis, and several candidate biological processes have been evaluated. (42). However, fibrosis is the outcome of fibroblast activation. Reversing the deposition of collagen and other proteins in ECM is often a challenge once fibrosis has occurred. Indirect evidence of collagen deposition or fiber formation can be derived from the evaluation of fibroblast activation. As a result, it determines when fibrosis can still be prevented and the course of the disease altered (43, 44). According to Stein S et al., constitutive deletion of FAP decreases experimental atherosclerosis progression and increases plaque stability with reduced collagen breakdown (45).

Our data showed higher SUV_mean_ uptake in non-calcific coronary artery territory, which may be a critical measurement in estimating the developmental stage of myocardial fibrosis. Since calcification is a late result of coronary plaques, using SUV value stratification can help identify coronary plaque activity and intervene in advance. Our cohorts’ data showed that SUV_mean_ has certain diagnostic efficacy in distinguishing calcified and non-calcified plaques. Due to the focal type of FAPI uptake in most myocardium, SUV_max_ values in other myocardium uptake regions were near the low level of background, except for the higher SUV_max_ values in the myocardium uptake regions. SUV_mean_ values seem to reflect the overall FAPI uptake of the myocardium in the case of low FAPI uptake in most myocardial regions. Studies in combination with coronary angiography or CTA examination should be intensified in the future.

### Limitations

This study has several limitations. An analysis of FAPI imaging was conducted on a small group of individuals with varying levels of cardiovascular risk, limiting the interpretation of statistical relationships between PET parameters, biomarkers, and LVEF. However, the study is a retrospective pilot investigation on a heterogeneous group of patients, allowing for a more accurate assessment of fibroblast activation under different circumstances. Our patients typically had multivessel coronary artery disease, which presented another limitation. Since not all patients underwent coronary CTA or coronary arteriography, this study cannot determine the extent to which other non-culprit stenoses influenced the tracer uptake. There is no comparison with alternative imaging methods of cardiac fibrosis, such as MRI, since the complementary anatomical, functional and molecular information provided by hybrid systems can simplify the evaluation procedure of various pathologies in a routine clinical setting. Subsequent more detailed population classification and large sample study is the direction of research.

## Conclusion

Al^18^F-NOTA-FAPI PET imaging can reflect the process of myocardial fibrosis. FAPI PET imaging is helpful for early intervention and treatment in patients at elevated risk of CVD, especially in patients with diabetes, obesity, and the elderly. Meanwhile, the combination of FAPI PET imaging with coronary artery CTA, nuclear magnetic myocardial perfusion imaging, echocardiography, and other non-invasive examination methods may significantly improve the accuracy of the assessment of myocardial fibrosis degree. Finally, applying different parameters of FAPI PET imaging such as SUV_max_, SUV_mean_, SUV_peak,_ and TBR to evaluate myocardial fibrosis is still worthy of further investigation.

## Data availability statement

The original contributions presented in this study are included in the article/Supplementary material, further inquiries can be directed to the corresponding author/s.

## Ethics statement

The studies involving human participants were reviewed and approved by the Ethics Committee of The First Affiliated Hospital of Harbin Medical University (approval no. 2021XJSS01). The patients/participants provided their written informed consent to participate in this study.

## Author contributions

ZL and WH were the guarantors of the integrity of the entire study. QS and SY contributed to the literature research. HZ and YJ contributed to the statistical analysis. CZ, PX, and YW contributed to the manuscript editing. All authors contributed to the study concepts/study design or data acquisition or data analysis/interpretation, manuscript drafting or manuscript revision for important intellectual content, approved the final version of the manuscript to be submitted, and agreed to ensure any questions related to the work are appropriately resolved.

## Footnotes

1
http://www.r-project.org

## Supplementary material

Supplementary Figure 1 The ROC curve for predicting calcified plaques by myocardial FAPI uptake (SUV_max_) in LAD, LCX, and RCA territory showed areas under the curve (AUCs) were 0.633 (95%CI: 0.365–0.9), 0.565 (95%CI: 0.294–0.835), and 0.537 (95%CI: 0.269–0.805), respectively. **(B)** The ROC curve for predicting calcified plaques by myocardial FAPI uptake (SUV_TBR_) in LAD, LCX, and RCA territory showed areas under the curve (AUCs) were 0.663 (95%CI: 0.414–0.913), 0.565 (95%CI: 0.308–0.822), and 0.63 (95%CI: 0.358–0.902), respectively.
