# Supplementary figures and images for: A clinical study on relationship between visualization of cardiac fibroblast activation protein activity by Al18F-NOTA-FAPI-04 positron emission tomography and cardiovascular disease
Source: Front Cardiovasc Med. 2022 Aug 22;9:921724. doi: 10.3389/fcvm.2022.921724 (PMC9441604; doi:10.3389/fcvm.2022.921724)

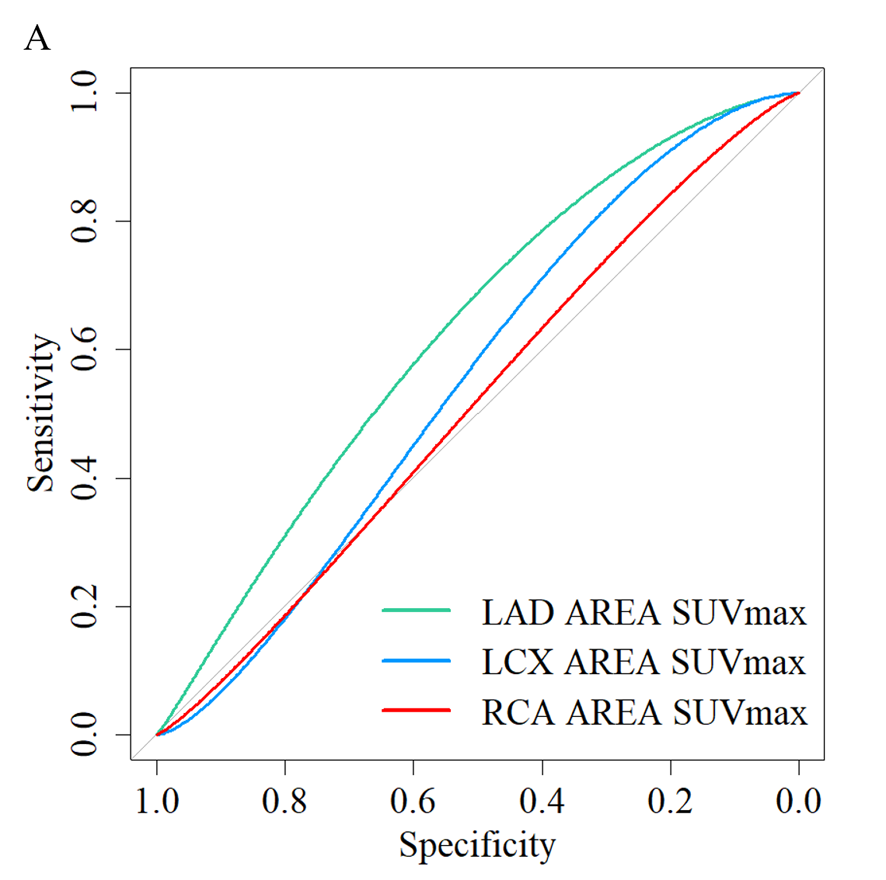

Supplement: Supplementary Figure 1 — The ROC curve for predicting calcified plaques by myocardial FAPI uptake (SUVmax) in LAD, LCX, and RCA territory showed areas under the curve (AUCs) were 0.633 (95%CI: 0.365–0.9), 0.565 (95%CI: 0.294–0.835), and 0.537 (95%CI: 0.269–0.805), respectively. (B) The ROC curve for predicting calcified plaques by myocardial FAPI uptake (SUVTBR) in LAD, LCX, and RCA territory showed areas under the curve (AUCs) were 0.663 (95%CI: 0.414–0.913), 0.565 (95%CI: 0.308–0.822), and 0.63 (95%CI: 0.358–0.902), respectively. [file Image_1.TIF]

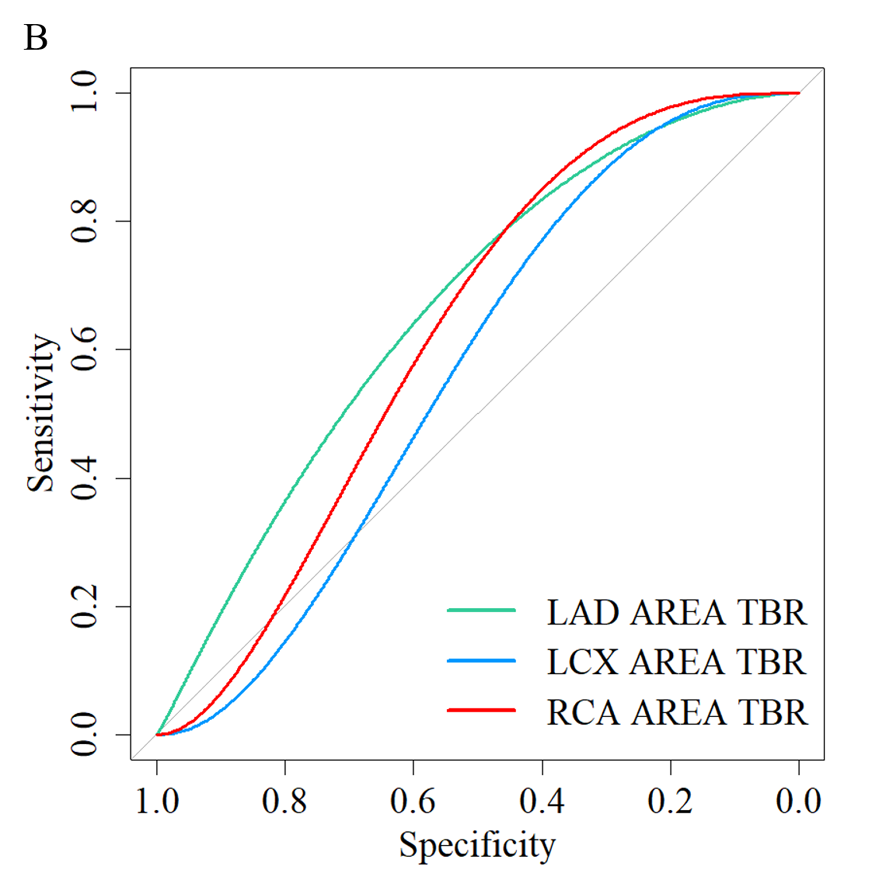

Supplement: Supplementary file 2 [file Image_2.TIF]
